# The effect of prebiotic fibre on the gut microbiome and surgical outcomes in patients with prosthetic joint infection (PENGUIN) - study protocol for a randomised, double-blind, placebo-controlled trial (ACTRN12623001273673)

**DOI:** 10.1186/s12937-024-01034-z

**Published:** 2024-10-25

**Authors:** Deepti K. Sharma, Balamurugan Ramadass, Stuart A. Callary, Anthony Meade, Rishikesh Dash, Robyn Clothier, Gerald J. Atkins, L. Bogdan Solomon, Boopalan Ramasamy

**Affiliations:** 1 Department of Orthopaedics and Trauma, Royal Adelaide Hospital, Adelaide, 5000 Australia; 2 Centre for Orthopaedic and Trauma Research, University of Adelaide, Adelaide, 5000 Australia; 3 Centre of Excellence for Clinical Microbiome Research (CCMR), All India Institute of Medical Sciences, Bhubaneswar, Odisha India; 4 Central Northern Adelaide Renal and Transplantation Service, Royal Adelaide Hospital, Adelaide, Australia

**Keywords:** Prosthetic joint infection, Gut dysbiosis, Resistant starch, Prebiotic fibre, Two-stage revision, Arthroplasty, Zonulin levels

## Abstract

**Background:**

Prosthetic Joint Infection (PJI) is the most devastating complication of arthroplasty surgery and affects 1–5% of patients. Despite strict adherence to aseptic protocols and preventive measures, infection is the most common reason for revision arthroplasty, and the incidence is increasing. Treatment of PJI is challenging and often requires repeated major surgeries with sequentially poor results. The continued occurrence of PJI, and persistence after treatment, brings into question the current treatment paradigm. Preclinical evidence suggests a link between altered gut health and the risk of PJI in arthroplasty patients. Resistant starches helps to restore gut physiology by enhancing the beneficial microbiome and producing short-chain fatty acids, which have several health-conferring properties. The primary aim of this study is to investigate the effect of a commercially available prebiotic fibre formulation on the gut microbiome in PJI patients planned for a two-stage revision surgery.

**Methods:**

A double-blind placebo-controlled trial will assess the effect of 8-week supplementation of a commercially available prebiotic supplement in patients presenting with first-time PJI undergoing two-stage revision surgery. The supplementation phase will start after the first stage revision, and 80 patients will be randomised to receive either a test product (34 g of resistant starch) or a placebo (custard powder) daily for eight weeks. Stool and blood specimens will be collected at baseline, four weeks and eight weeks after the first-stage surgery and once at second-stage surgery. Gut microbiome profile, inflammatory cytokines and gut permeability biomarkers will be measured. Tissue specimens will be collected intra-operatively during first and second-stage surgeries. Baseline dietary patterns and gut symptoms will be recorded using validated questionnaires. Treatment outcomes will be reported for both cohorts using the Delphi criterion at one and two years after second-stage surgery.

**Discussion:**

This will be the first study to investigate the relationship between gut health optimisation and preventing PJI recurrence in arthroplasty patients. If supplementation with resistant starch improves gut health and reduces systemic inflammation, optimising the gut microbiome will be a recommended preoperative management strategy for arthroplasty patients.

**Trial registration no:**

ACTRN12623001273673.

**Supplementary Information:**

The online version contains supplementary material available at 10.1186/s12937-024-01034-z.

Arthroplasty or joint replacement is a life-changing surgery for millions of people with end-stage arthritis. Successful joint replacement provides pain relief and restores function and independence, significantly improving the patient’s quality of life. With the increasingly aged population, longer life expectancy and lifestyle factors, the demand for joint replacement is increasing exponentially. By 2030, the rate of knee and hip replacement surgeries in Australia is predicted to increase by 276% and 208% respectively [1]. While arthroplasty is generally successful, prosthetic joint infection (PJI) is a catastrophic complication. As per the latest Australian Orthopaedic Association National Joint Replacement Registry (AOANJRR) report, infection is the leading cause of revision surgery in Australia, with 51% of hip revisions and 24% of knee revisions occurring due to infection within three months of the primary surgery [2]. PJI patients have a substantial risk of early death. In Australia, the five-year survival of these patients is just 79%, which is worse than that of many primary cancers, including thyroid, prostate, breast, melanoma, and uterine cancers [3], while the 10-year survivorship is 60% [4]. In a US study, revision arthroplasty for PJI was associated with a fivefold increase in mortality compared with revision arthroplasty for aseptic failures [5]. The quality of life in PJI patients is reported to be worse than in patients with debilitating strokes [6].

Surgery is the most effective option to treat PJI. The Prosthetic Joint Infection in Australia and New Zealand Observational (PIANO) study, which is the largest, multicentre study involving 27 Centres across Australia and New Zealand (including our hospital), reported a 46% failure rate to cure PJI within 24 months using the current standard of care [7]. The surgeries are high-risk, resource-intensive and often require repeated major surgeries. With every admission, the outcomes are increasingly poor, with a higher rate of in-hospital mortality accompanied by inordinate costs that threaten healthcare systems [8–11]. Infection from revision hip replacement has more significant mortality than hazardous procedures such as coronary interventions, carotid surgery, kidney transplants and aseptic arthroplasty revision [12]. In a recent analysis of Australian patients undergoing two-staged revision for PJI cost of treatment was found to be $113,226 at a two-year follow-up [13]. Despite implementing all known patient optimisation and presurgical strategies to reduce PJI, particularly *Staphylococcus aureus* at the surgical site, PJI still occurs [14–17]. Hence, there is an urgent clinical requirement to investigate new preventive strategies to reduce the chances of PJI.

Emerging evidence suggests that the gut microbiome - a range of microorganisms that inhabit the gastrointestinal tract, including bacteria, fungi, viruses, and their genetic components, may play a role in the pathogenesis of musculoskeletal infections [18]. The gut microbiome maintains a homeostatic equilibrium in healthy individuals and resists perturbations. Several factors such as diet, antibiotics, lifestyle, age, gender, compromised immune system, and host genetics can affect the homeostatic configuration of gut microbiota, resulting in a state referred to as dysbiosis [19] or leaky gut, which has been linked to a variety of health disorders, including PJI [18]. Gut dysbiosis can result from either the absence or reduced abundance of beneficial microorganisms, increased abundance of pathogens, altered metabolic functions, or reduced diversity [19]. Preclinical evidence generated using a mouse model representative of clinical PJI [20] showed that susceptibility to PJI was increased by almost 50% in mice with a disrupted gut microbiome before surgery [18]. Furthermore, those mice with a disrupted gut microbial community that went on to develop PJI showed a reduced systemic immune response to infection measured by serum markers and immune cell populations in the spleen [18]. Hence, in the second International Consensus Meeting on Musculoskeletal Infection (MSIS), the need to focus on the gut microbiome as a modifiable factor to treat or reduce the incidence of PJI has been highlighted [21].

Gut dysbiosis is a modifiable condition that can be corrected with appropriate dietary interventions. Profound changes in the gut microbiome can be rapidly achieved by macronutrient changes such as a high-fibre diet or by adding prebiotics or probiotics. A previously published study has demonstrated that a high-fibre diet can alter the gut microbiome favourably in just 24 hours [22]. Probiotics are live microorganisms that confer health benefits when administered in adequate amounts. In contrast, prebiotics are non-digestible fibres that pass through the gastrointestinal tract undigested and are fermented in the colon to produce short-chain fatty acids (SCFAs), such as acetate, propionate and butyrate. These SCFAs, in turn, stimulate the growth and activity of ‘beneficial’ bacteria in the large intestine and have independent health-conferring properties [23, 24].

Resistant Starch (RS) is a type of prebiotic fibre that is found in some legumes and grains (RS 1,2) or produced during the cooking process (RS3) or can be created commercially through the process of chemical cross-linking such as high amylose corn starch (RS 4). Numerous high-amylose corn starches (RS2) are commercially available and can be incorporated easily into the diet to improve gut health. Clinical studies have shown that feeding RS can alter the gut microbiota favourably and reduce the incidence of several diseases [25–29]. Philips et al., in 1995 [30] demonstrated that intake of 39 g resistant starch for three weeks significantly improved the gut microflora and short-chain fatty acid production. Probiotics or prebiotics alone or in combination (synbiotics) have been shown to significantly improve surgical outcomes in liver transplant patients who are at a very high risk of infection, organ rejection and death and in patients undergoing major abdominal surgery [31]. Supplementation was associated with a significantly reduced infection rate, fewer antibiotics prescribed, shorter hospital stays and lower incidence of other complications [31, 32]. These data suggest that adding a prebiotic or probiotics as an adjuvant therapy to the antibiotic regimen may help treat PJI patients more effectively. None of the clinical studies have investigated whether dietary interventions can alter the gut microbiome to improve surgical outcomes in PJI patients. Our study will be the first proof-of-concept study with an overarching objective of identifying a new treatment paradigm for PJI patients. The preliminary evidence linking gut dysbiosis to the incidence of PJI suggests that the outcomes of this study may provide a novel treatment method for this medical condition.

## Study aim

The primary aim of this study is to investigate the effect of a commercially available prebiotic fibre formulation on the gut microbiome in PJI patients planned for a two-stage revision surgery. The secondary aims include the impact of supplementation on blood, tissue and stool biomarkers after 8 weeks of supplementation and treatment outcomes following second-stage revision surgery based on the Delphi criterion.

## Hypothesis

We hypothesise that resistant starch supplementation will improve gut health and treatment outcomes in PJI patients.

## Methods

### Participants

Patients scheduled to undergo two-stage revision for the treatment of PJI at the Royal Adelaide Hospital between January 2024- December 2025 will be recruited for this study. The study has been approved by the Central Adelaide Local health Network Human Research Ethics Committee (CALHN HREC) and all participants will provide written informed consent. The trial has been registered with the Australian and New Zealand Clinical Trials Registry (ACTRN12623001273673).

### Inclusion criteria

Patients will be included if they meet all of the following criteria:

- Adult patients with a first-time diagnosis of PJI as per the MSIS criteria and scheduled to undergo two-stage revision at the Royal Adelaide Hospital;
- Willingness to participate in the study;
- Sufficiently healthy to undergo two-stage revision surgery.

### Exclusion criteria

Patients will be excluded if they meet any of the following exclusion criteria:

- Patients with chronic PJI;
- Patients who do not consent to the study;
- Pregnant and breast-feeding women;
- Patients who cannot tolerate prebiotic fibre;
- Patients who are immunosuppressed and on dietary restrictions.

#### Study Design and products

**Fig. 1:**
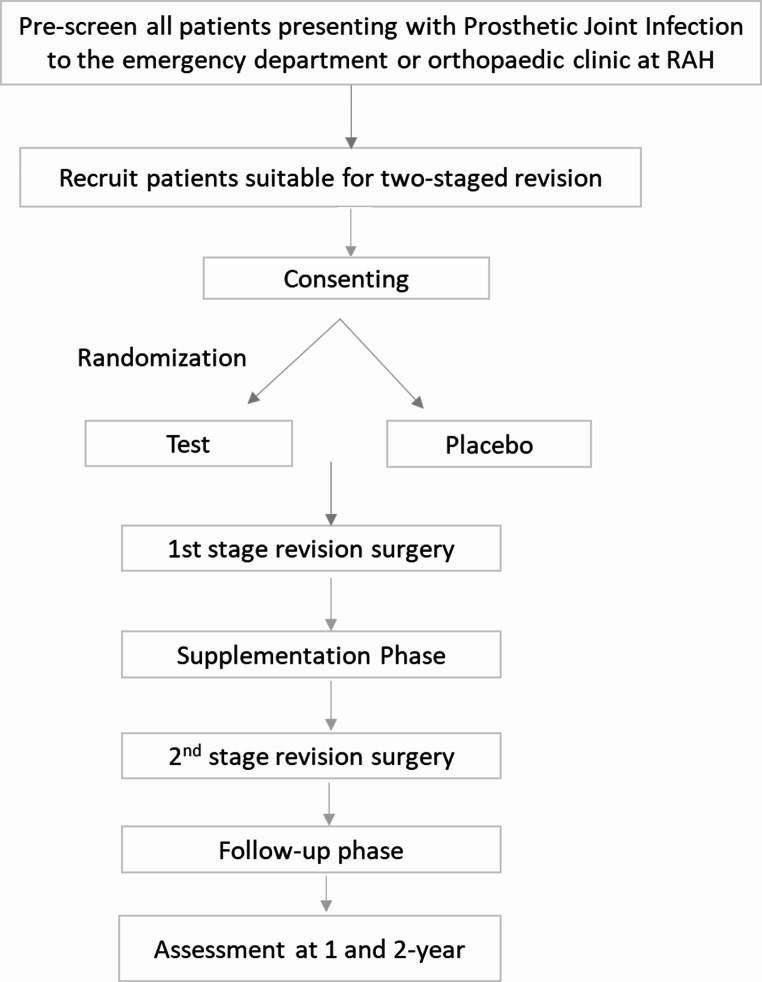
PENGUIN Study flowchart

In this 8-week, single-centre, double-blind, randomised controlled trial, 80 patients scheduled to undergo two-stage revision surgery for the treatment of PJI will be randomised to receive resistant starch or a placebo containing corn starch. The test product is a commercially available ready-to-mix beverage powder rich in resistant starch (34 g high amylose corn starch, RS2, per serve) available in the Australian market. Patients in the intervention group will receive one sachet of the test product, and those in the placebo group will receive an equivalent amount of ready-to-mix corn-based custard powder for eight weeks. The test and the placebo products will be procured from the market and re-packed in unlabelled plain sachets in a Food Standards Australia New Zealand (FSANZ) approved facility. The treating surgeons will recruit the participants when they present to the Department of Orthopaedics and Trauma for PJI treatment. All patients will be followed up at 2, 4 and 8 weeks after first stage revision and one and two years after the second-stage revision. The study flowchart is shown in Fig. 1.

### Treatment allocation, randomisation and blinding

Patients who consent to participate will be enrolled in the study and assigned a code. Once enrolled, all patients will undergo standard-of-care treatment for their PJI, including optimisation by a multidisciplinary team comprising an anaesthetist, infectious diseases consultant, orthopaedic doctors, joint replacement nurses, dietitian, and post-surgical antibiotic therapy. Using a computer-generated block randomisation plan, one study investigator will randomly assign the patients to the placebo or intervention group. The treating surgeon and the patient will be blinded to the treatment allocation. Supplementation will start soon after the first-stage revision surgery when the patient is well enough to take oral foods. Based on the randomisation plan, the recruited patients will receive one sachet of test or control product mixed with milk, juice or food of their choice once a day for eight weeks. All in-patients will get the product with breakfast, supervised by the study dietitian. On discharge, patients will be given the clinical supplies to complete the supplementation daily for eight weeks. One of the investigators will ensure compliance through regular phone calls.

### Outcomes measures

Primary outcome: The primary outcome is the change in gut microflora between the placebo and the test group after 8 weeks of supplementation.

Secondary outcomes: The study has several secondary outcomes, which are listed below:

1. Change in faecal calprotectin and short-chain fatty acids within and between cohorts at the end of 8-week supplementation.
2. Change in Interleukin levels (IL-1, IL-6 and IL-10) within and between cohorts at the end of 8-week supplementation.
3. Changes in C-reactive protein, CD4 and zonulin levels within and between the placebo and the test group at the end of 8-week supplementation.
4. Tissue microbiology results will be compared between cohorts during first and second-stage surgery.
5. Treatment outcomes using the Delphi criterion [33] (supplementary file 1) will be reported for the two cohorts at one and two years after second-stage surgery.

### Data collection

**Fig. 2:**
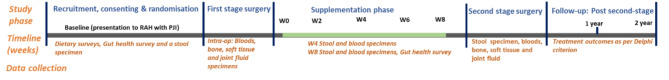
Schematic representation of study design and data collection The green line represents the supplementation period

The timeline of the follow-up visits and data collection is presented in Fig. 2, and the timeline for measurements is given using The Standard Protocol Items: Recommendation for Interventional Trials (SPIRIT) diagram in Table 1. If any participant fails to continue the study protocol or has less than 80% compliance, the data will be recorded but not included in the final analyses. The results will be presented per protocol as recommended for nutrition studies [34].

#### Stool samples

Stool specimens will be collected on four separate occasions: after the first-stage revision surgery (baseline, t0), at four weeks (t4) and eight weeks (t8) post-supplementation. The fourth sample will be collected at the time of second-stage surgery. Participants will be asked to store the specimens in a freezer (sealed in the stool kits provided) and bring them along on the day of the clinical review. All specimens will be stored at -80 °C until further analysis. The modified Gastrointestinal Symptom Rating Scale Questionnaire (mGSRS) will be administered to assess the overall gut symptoms of participants. The mGSRS is a short series of questions about gastrointestinal symptoms. Additional questions about stool frequency (how often), consistency (using Bristol Stool Chart) and perceived normality of bowel habits (Likert scale) are included (supplementary file 2). The gut health survey will be done at baseline (t0) and 8-week time-point (t8). Stool DNA will be extracted per the previously published protocols [35]. Real-time polymerase chain reaction will assess the abundance of known enteric pathogens. Deep sequencing of the DNA will be done to determine the nature of the microbiota. The gut microbiome will be analysed using the R Package, and the abundance of microbes, including phylum, genus and species level, will be correlated with PJI. Faecal inflammation biomarkers, including faecal calprotectin, will be measured with ELISA. Short-chain fatty acids and pH will be measured as published previously [36, 37].

**Table 1:**
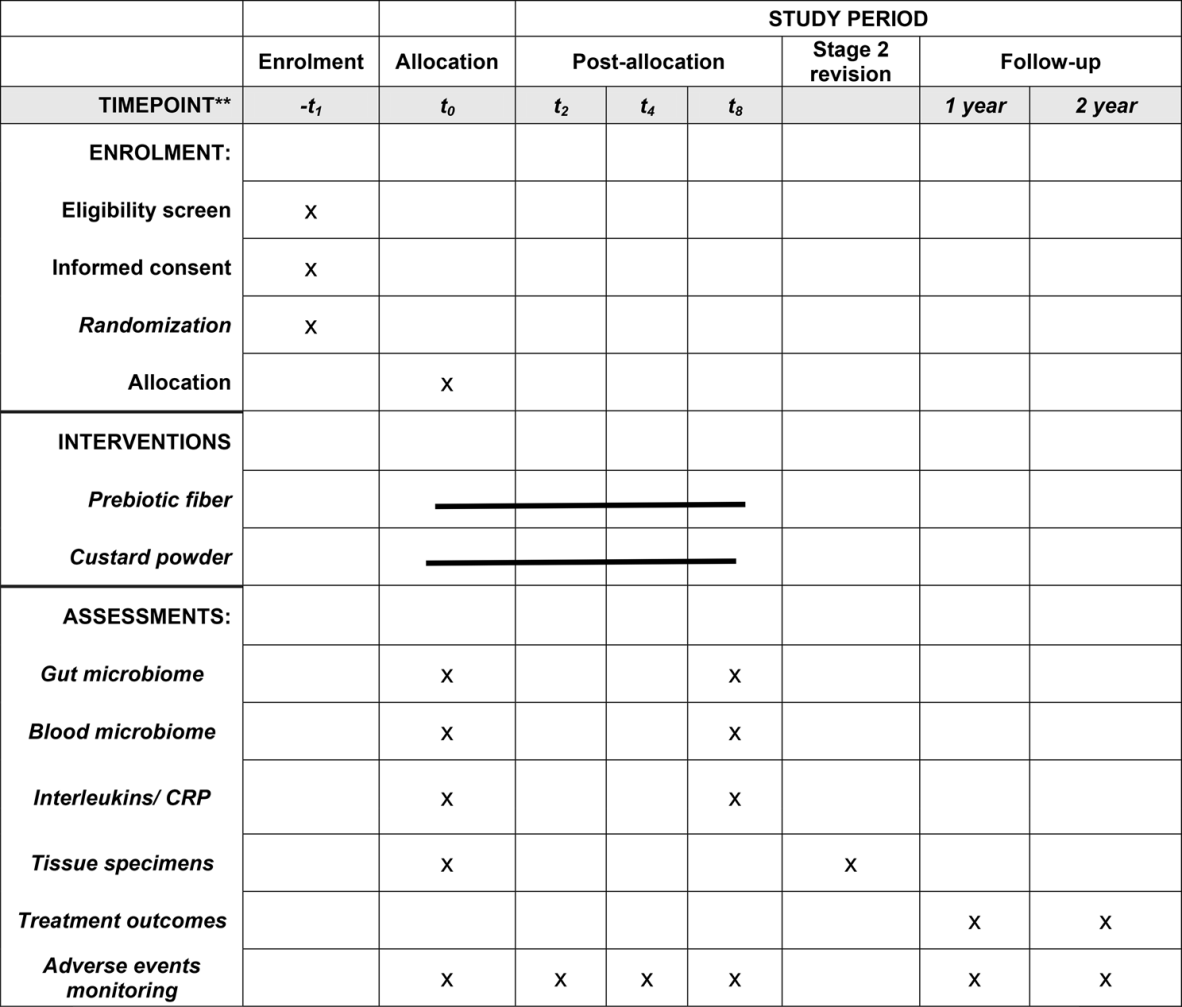
Schedule of enrolment, interventions, and assessments

#### Blood and bone tissue collection

All tissue samples will be taken during the first-stage and second-stage surgeries. Soft tissue, bone, and synovial fluid (when present) will be retrieved intra-operatively using sterile surgical instruments and snap-frozen immediately upon collection. Identifiers will be removed, and the samples will be labelled with the study ID code. During each surgery, 10 ml of venous blood will be collected in SST, heparin and EDTA-coated tubes intraoperatively. The specimens will be processed, split in Eppendorf tubes and snap-frozen at -80 °C until further analysis. The plasma and serum specimens will be analysed for inflammatory markers, including interleukins (IL-1, IL-6 and IL-10), C-reactive protein (CRP), erythrocyte sedimentation rate (ESR), albumin, CD4/CD8, CD14, lipopolysaccharides (LPS), Zonulin and tumour necrosis factor-alpha (TNF-alpha) using validated ELISA assays. Blood DNA will be extracted from whole blood, and deep sequencing of the DNA will be done to determine the nature of the microbiota [38]. The blood microbiome will be analysed using the R Package, and the abundance of microbes, including phylum, genus and species level will be compared between the groups.

#### Dietary pattern

Patients’ dietary patterns at baseline will be collected using a food frequency questionnaire screener (FFS) validated for the Australian population and a 4-day Food Diary by the study dietitian (AM). The patients who habitually consume a higher-fibre diet may have a significantly different microbiome profile than those who consume a lower-fibre diet. Hence, this tool will allow us to identify the added effect of prebiotic fibre.

#### Patient data

The participant’s medical records will be accessed to extract information on previous total joint replacement, demographics, medical and surgical history, physical examination, laboratory investigations, medical imaging, operative reports, and readmission due to infection and surgical complications. This information is a part of the standard of care protocol for any PJI patient, independent of study participation.

#### Assessment of treatment outcomes based on the Delphi criterion

The longer-term outcome of this study will be to investigate treatment success based on the Delphi criterion for PJI following second-stage revision surgery. The Delphi group treatment success is based on three critical dimensions: (1) eradication of infection, (2) no subsequent surgical intervention, and (3) no mortality related to PJI (supplementary file 1) [33]. The time point followed will be one and two years after the second stage of surgery.

### Adverse outcomes

Patients will be instructed to inform the researchers about any changes or discomfort experienced with the products. However, they will be counselled to help understand normal expectations when starting a prebiotic, e.g., flatulence for a few days. Furthermore, during follow-up visits, the researchers will ask about adverse events which may be related to the intervention. Adverse events, if any, will be recorded and reported in publication. The likelihood of adverse events after administering the test or placebo products is negligible, as both are well-established in-market products.

### Ethical approval

The Central Adelaide Local Health Network Human Research Committee (CALHN-HREC) has approved the study (Approval No. 2023HRE00206). All participants will give informed consent before enrolment and randomisation. The treating surgeons or registrars will explain the information about the trial.

### Clinical trial registration

The study has been registered in the Australia New Zealand Clinical Trials Registry, and the trial number is ACTRN12623001273673.

### Statistical analysis

There are no studies investigating the improvement in gut microflora in patients with joint infections. Hence, we have based our sample size calculation on the superiority test. We assume a 10% difference in beta diversity between the groups will be a clinically meaningful change. Hence, for a mean difference of 0.1 with an assumed standard deviation of 0.075 at a 5% significance level and accounting for a 10% dropout rate, 80 patients will be required to demonstrate significant changes between the groups at 8 weeks. Normality will be assessed using the Shapiro-Wilk test. Mean and standard deviation or median and interquartile range will be reported. Paired t-test or Wilcoxon test will assess within-group differences, and an Independent t-test, or Mann-Whitney will assess the between-group differences. The secondary outcome measures investigating the outcomes from stool, blood and tissue specimens will be compared between the placebo and test cohorts using a paired t-test or Wilcoxon test for within-group differences and an Independent t-test or Mann-Whitney to investigate between-group differences. Results will be analysed on IBM SPSS 27.

## Discussion

To the best of our knowledge, this is the first randomised, double-blind, placebo-controlled trial to assess the effect of resistant starch in correcting gut dysbiosis to improve treatment outcomes in PJI patients.

The incidence of PJI is increasing worldwide. The success rate of treating PJI ranges from 0 to 89%, with the highest treatment success achieved in patients who received treatment within 30 days of onset, were infected with low virulence microorganisms and were relatively healthy at baseline [39]. A high level of culture negativity and the presence of polymicrobial PJI make this condition very challenging for orthopaedic surgeons to treat. Given its grave prognosis and lack of an established treatment protocol, investigating preventive strategies is an important clinical requirement.

The increasing rates of PJI suggest that the current modes of prevention are inadequate, and other preventive strategies need to be investigated. Preliminary evidence from animal models and pilot data generated at our hospital suggests that PJI patients suffer from gut dysbiosis. Changes in the macronutrients, such as a high-fibre diet, can correct gut dysbiosis. The health benefits of high amylose-resistant starch in altering colonic physiology have been well documented. High amylose-resistant starch is readily fermented in the human gut, significantly changing luminal pH and short-chain fatty acid concentrations. Furthermore, resistant starches can modulate adaptive and innate immune responses, which may reduce the chance of biofilm formation and implant-associated infections in PJI patients directly by increasing the concentration of beneficial bacteria and producing short-chain fatty acids, thus improving patient outcomes.

To our knowledge, only one clinical study has investigated gut dysbiosis directly in PJI patients. This recent study reported significantly (*p* < 0.001) higher levels of Zonulin, a marker of increased gut-permeability, in PJI patients (7.6 ± 6.1 ng/ml) as compared to primary arthroplasty patients (3.9 ± 4.5 ng/ml) [40]. While this study used surrogate markers of gut permeability, pilot data generated at our hospital (unpublished) suggests the existence of gut dysbiosis in PJI patients. In the current study, we will administer resistant starch or placebo to PJI patients for eight weeks and investigate the changes in the gut microbiome. In addition, an array of plasma inflammatory and gut permeability markers and blood microbiomes will be measured. Apart from directly assessing gut dysbiosis, this study will also evaluate if the dysbiosis can be reset through prebiotic intervention.

There are some limitations in the design of this study. Firstly, this is a single-centre study with an estimated sample size. It is clinically and ethically essential to generate proof of concept data before any large-scale interventions can be planned. If the results from this initial study are promising, we will undertake a multicentre study to validate the findings. Secondly, the impact of change in gut microbiome on surgical outcomes will be evaluated one and two years after the second stage revision. Due to the nature of PJI, many patients may not be able to make it to the longer-term follow-up either due to premature death or non-resolution of infection, thus requiring several repeat first-stage surgeries, or may not be medically fit to undergo second-stage revision due to comorbidities. Lastly, the patients will take the product at home (after discharge) and administer the intervention products. To encourage compliance, one of the study investigators will phone the patients regularly and empty packages will be returned to investigators.

In conclusion, this study’s findings will provide the first clinical evidence of targeting gut health to prevent and manage joint infections.

## Electronic supplementary material

Below is the link to the electronic supplementary material.

Supplementary Material 1

Supplementary Material 2

## Data Availability

No datasets were generated or analysed during the current study.
