# Supplementary material for: The effect of prebiotic fibre on the gut microbiome and surgical outcomes in patients with prosthetic joint infection (PENGUIN) - study protocol for a randomised, double-blind, placebo-controlled trial (ACTRN12623001273673)
Source: Nutr J. 2024 Oct 25;23:132. doi: 10.1186/s12937-024-01034-z (PMC11515416; doi:10.1186/s12937-024-01034-z)
Supplement: Supplementary file 1 — Supplementary Material 1 [file 12937_2024_1034_MOESM1_ESM.docx]

**Delphi Criterion for Treatment Success**

| **Dimension A**  **Infection eradication, characterized by:** | No clinical failure (healed wound without fistula or drainage and painless joint)  Return to baseline (or to the normal level) of serologic markers (including C-reactive protein, erythrocyte sedimentation rate, white blood cells)  No need for antibiotic suppression treatment  No infection recurrence caused by the same organism strain  No incident (new) infection with a different organism  No concomitant PJI in other surgical site |
| --- | --- |
| **Dimension B**  **No subsequent surgical intervention after reimplantation surgery, characterized by:** | No reoperation attributable to infection  No revisions owing to aseptic causes in the proposed follow-up period (including aseptic loosening, instability, stiffness)  No radical surgeries after reimplantation (including: lower limb amputation, hip/ knee arthrodesis, hip resection arthroplasty) |
| **Dimension C**  **No presence of PJI-related morbidity or mortality, characterized by:** | No death caused by a condition directly linked to PJI (including sepsis, necrotizing fasciitis).  No death in a hospitalization owing to treatment of PJI, by causes not directly linked with PJI (including myocardial infarction, stroke, pneumonia)  Hospital length of stay less than 3 months/year during treatment of PJI  No medical complications associated with PJI intravenous or local antibiotic treatment (including systemic toxicity, renal insufficiency, peripherally inserted central catheter line complications) |
