# Supplementary material for: The effect of prebiotic fibre on the gut microbiome and surgical outcomes in patients with prosthetic joint infection (PENGUIN) - study protocol for a randomised, double-blind, placebo-controlled trial (ACTRN12623001273673)
Source: Nutr J. 2024 Oct 25;23:132. doi: 10.1186/s12937-024-01034-z (PMC11515416; doi:10.1186/s12937-024-01034-z)
Supplement: Supplementary file 2 — Supplementary Material 2 [file 12937_2024_1034_MOESM2_ESM.docx]

**Modified Gastrointestinal Symptom Rating Scale Questionnaire**

| On average, over the past WEEK, how often did you open your bowels? | | | | | | | |
| --- | --- | --- | --- | --- | --- | --- | --- |
| Less than once every 4 days | | | Once every 3-4 days | Once every 1-2 days | 1-2 times a day | 3-5 times a day | >6 times a day |
| Please refer to the Bristol Stool Chart attached and tick the answer which best describe(s) the stool you passed in the past WEEK: | | | | | | | |
| 🞎  🞎  🞎  🞎  🞎  🞎  🞎  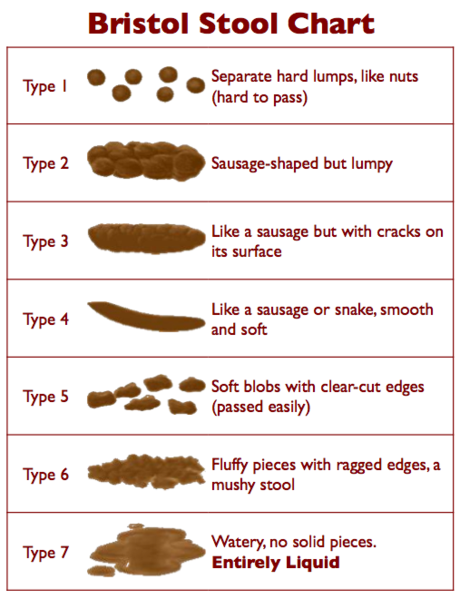 | | | | | | | |
| Have you used any of these products or medications in the past WEEK? | | | | | | | |
| Y | N | Probiotics (e.g. Yakult, Inner Health Plus, VSL #3)? | | | | | |
| Y | N | Stool softeners such as Coloxyl, Senna, Lactulose (Duphalac), Movicol,  Liquid Paraffin (Parachoc), Sorbitol (Sorbilax) | | | | | |
| Y | N | Antibiotics (e.g. ciprofloxacin, vancomycin, flucloxacillin, Augmentin, clindamycin, Linezolid, fluconazole) | | | | | |
| Y | N | Loperamide (e.g. Gastro-Stop, Imodium) | | | | | |
